# The Web-based Open-access Reliable Decision on Synonyms (WORDS) English Vocabulary Test

**DOI:** 10.5334/joc.391

**Published:** 2024-07-29

**Authors:** Po-Heng Chen, Rachael C. Hulme, Lena M. Blott, Jennifer M. Rodd

**Affiliations:** 1 Department of Experimental Psychology, University College London, London, United Kingdom; 2 Graduate Institute of Linguistics, National Taiwan University, Taipei, Taiwan; 3 Centre for Applied Behavioural Sciences, Department of Psychology, School of Social Sciences, Heriot-Watt University, Edinburgh, United Kingdom; 4 Department of Psycholinguistics, School of Humanities, University of Mannheim, Mannheim, Germany

**Keywords:** vocabulary test, web-based, online, lexical semantics, item response theory

## Abstract

A quick and reliable test of vocabulary knowledge is a vital component of many studies looking at a range of language processing skills. Recent proliferation of online (web-based) research has generated a growing need for reliable open-access vocabulary tests that can be administered online. This data report presents the newly developed 30-item Web-based Open-access Reliable Decision on Synonyms (WORDS) English Vocabulary Test. In Experiment 1, we tested 109 participants (age range: 18–69 years) on an initial set of 51 potential multiple-choice test items in which participants read a target word (e.g., *ubiquitous*) and selected a (near-)synonym (e.g., *omnipresent*) from among three semantically unrelated foils (e.g., *interpersonal, catatonic, voluminous*). We conducted an item response theory analysis of participants’ accuracy data to select an optimal subset of 30 items to include in the final version of the test. In Experiment 2, we verified the reliability of this 30-item version in a different sample (*N* = 121; 18–79 years); reliability (internal consistency) was good (Cronbach’s α = 0.82). We confirmed that, consistent with numerous previous studies, (1) responses were more accurate and quicker for more frequent compared to less frequent words, and (2) older adults showed greater vocabulary knowledge compared to younger adults. The WORDS test takes on average 4 minutes (5 minutes 40 seconds including consent/debrief) to complete. It can be freely accessed via Gorilla Open Materials (https://app.gorilla.sc/openmaterials/694887), allowing ease of use and for useful comparisons between data collected by different researchers.

## Introduction

Successful language comprehension relies on good knowledge of words and their meanings (K. Nation, 2017; Perfetti, 2007; Perfetti & Hart, 2002). Vocabulary knowledge typically increases throughout adulthood, reflecting our ability to learn from our linguistic environment across the lifespan (Hoffman, 2018; Keuleers, Stevens, Mandera, & Brysbaert, 2015; Miller, Myers, Prinzi, & Mittenberg, 2009; Park et al., 2002; Verhaeghen, 2003). A reliable and valid test of vocabulary knowledge is vital for understanding the factors that drive vocabulary change and how this knowledge relates to a range of other important cognitive abilities. It can also be used as a quick screening task to ensure that participants have the necessary linguistic competence to complete tasks designed for native speakers (Rodd, 2024).

Individuals’ vocabulary knowledge can be assessed in several ways. First, researchers can simply ask yes/no questions about whether people know the meanings of words (e.g., The Eurocentres Vocabulary Size Test; Meara & Buxton, 1987). If researchers want to ensure that people know the *meanings* of words, they can ask them to give definitions or explanations (e.g., Biemiller & Slonim, 2001). More commonly, participants may be asked to match a given target word with one of several options (i.e., in a multiple-choice question) which can be displayed in various formats, such as pictures (e.g., The Peabody Picture Vocabulary Test; D. M. Dunn, 2019; L. M. Dunn & Dunn, 1997), definitions/descriptions (e.g., The Vocabulary Levels Test; Schmitt, Schmitt, & Clapham, 2001, and The Vocabulary Size Test; Nation & Beglar, 2007), or (near-)synonyms (e.g., The Mill Hill vocabulary scale; Raven, Raven, & Court, 1989). This approach probes vocabulary knowledge at the semantic level without relying on production skills. Using (near-)synonyms as the options of multiple-choice questions allows researchers to easily control the difficulty of the options using known psycholinguistic properties (e.g., word frequency, word length, etc). Although using multiple-choice questions is not without problems (e.g., random guessing and item-level sampling issues; see Gyllstad, Vilkaitė, & Schmitt, 2015 for detailed discussion), these drawbacks can feasibly be dealt by careful consideration of estimated item difficulty (Stoeckel, McLean, & Nation, 2020).

Advances in the tools for conducting online (web-based) psychological experiments (e.g., Anwyl-Irvine, Massonnié, Flitton, Kirkham, & Evershed, 2020; de Leeuw, 2015; Peirce et al., 2019) and crowdsourcing platforms (e.g., Amazon Mechanical Turk: https://www.mturk.com/; Prolific: www.prolific.com) have supported a rapid increase in web-based research in the past decade (see Rodd, 2024 for review). This shift from lab-based to web-based testing has generated a growing need for vocabulary tests that can be administered quickly and easily online. Translating traditional lab-based vocabulary tests (e.g., Baddeley, Emslie, & Smith, 1992; Raven et al., 1989) into web-based versions can be difficult due to copyright issues (e.g., Shipley, Gruber, Martin, & Klein, 2009), requirements for experienced administrators (e.g., Wiig, Semel, & Secord, 2013), long completion times (e.g., Brown, Fishco, & Hanna, 1993; I. S. P. Nation & Beglar, 2007; Wiig et al., 2013), or low reliability (e.g., Shipley, 1940; see Vermeiren et al., 2023 about the reliability of the Shipley test). Several online and freely accessible vocabulary tests for English native speakers are now available and deploy different formats from yes/no questions testing pure recognition of words (e.g., Meara & Miralpeix, 2016) to multiple-choice questions requiring differentiation between several definition/description options (e.g., Drown, Giovannone, Pisoni, & Theodore, 2023a; 2023b). A few self-administered and automated vocabulary tests with different formats (e.g., lexical decision, synonym matching, definition matching) have been demonstrated to have high correlations with another in-person vocabulary assessment requiring a trained administrator (the Peabody Picture Vocabulary Test) (Harel et al., 2024). Practically, using online self-administered vocabulary tests not only saves much time for both experimenters and participants, but also reaches samples with more diverse demographic characteristics (Rodd, 2024).

We aimed to contribute to this growing set of resources by developing a short, reliable, and open-access English vocabulary test that can easily be shared on a widely-used platform for running web-based experiments (e.g., Gorilla; Anwyl-Irvine et al., 2020) and to provide all materials and data on the Open Science Framework (OSF) for easy translation to other platforms. We assessed reliability (internal consistency) in a sample that spanned the adult lifespan to ensure that the test is suitable for all age groups and is not, for example, adversely affected by ceiling effects in older adults (Hoffman, 2018; Miller et al., 2009; Park et al., 2002; Verhaeghen, 2003).

To develop an optimal set of items we took a two-stage approach. In Experiment 1, we tested participants’ performance on a set of 51 potential target items that had been selected on the basis of their word frequency, aiming to span the range from easy (high-frequency, e.g., *collect*) to difficult (low-frequency, e.g., *penurious*) words. We then selected an optimal subset of items to be included in the final short test by conducting an item response theory (IRT) analysis on these data. IRT is a statistical approach for analysing test responses with the aim of improving measurement reliability. IRT uses a collection of statistical models to try to specify the relationship between person properties and item properties based on observed test responses (for detailed introductions to IRT, please see Baylor et al., 2011; Edwards, 2009).

This approach has previously been used to improve test reliability on a range of assessments of reading ability and vocabulary knowledge (Beglar, 2010; Vermeiren et al., 2023; Yeatman et al., 2021). For example, Yeatman et al. (2021) aimed to assess children’s reading ability. To reduce the number of items and find an optimised subset of items, they conducted an IRT analysis based on children’s accuracy on a lexical decision task, reducing their initial set of 500 items to produce three 76-item (38 real words and 38 pseudowords) lists with a good reliability (*r* ≥ 0.92) wherein items spanned the full range of difficulty to ensure children of different levels of reading ability could be assessed effectively.

Here, we closely followed the procedure set out by Yeatman et al. (2021) to analyse the accuracy data in Experiment 1 and derive an optimal subset of 30 words for use in Experiment 2. First, we correlated participants’ accuracy on individual items with their mean accuracy across all items. If an item was good at predicting vocabulary knowledge, then participants who responded correctly to this item ought to have higher mean accuracy across the entire set of items compared to those who did not. We therefore removed items that had a very low correlation coefficient (*r* < 0.1). We then applied two separate IRT models to this data set. First, we used a one-parameter logistic model (also known as the 1-PL or Rasch model), which only includes a single item-difficulty parameter (represented by *b*). By removing words where the fit between the data and the model was poor, we aimed to exclude any items with unpredictable responses. Second, we used a two-parameter (2-PL) logistic model, which additionally includes a discrimination parameter that captures the extent to which each item can discriminate between individuals of different abilities. Finally, we confirmed the effectiveness of our IRT analysis by testing the reliability (internal consistency) of the retained subset of 30 words.

In Experiment 2, we then tested the reliability (internal consistency) of the final version of the WORDS vocabulary test that included only the optimised set of 30 items in a new sample of 121 participants. Specifically, we required that the reliability (internal consistency) of this vocabulary test based on participants’ accuracy data should reach at least a ‘good’^1^ level (i.e., Cronbach’s α ≥ 0.8). In addition, we assessed the validity of the test by confirming the presence of well-established effects of word frequency and age on participants’ performance.

## Experiment 1

Experiment 1 aimed to select an optimal set of words for the final vocabulary test from an initial set of 51 potential items using an IRT analysis of participants’ accuracy data. Materials, data, and analysis scripts for Experiment 1 are available on the OSF: https://osf.io/mgsdq/. (This experiment also included an additional 52 trials in which the target words were high ambiguity words, e.g., *fan*. These trials were included to address a separate research question. The raw data for these items are included on the OSF for completeness but analysis of these items is not reported here.)

### Method

#### Participants

We recruited 114 participants (aged 18–69) via Prolific (www.prolific.co) with the following criteria: (1) aged 18–69 years, (2) located in the UK, (3) British English as first language, (4) no diagnosis of dyslexia or another reading or language disorder, (5) no diagnosis of mild cognitive impairment or dementia, (6) no visual impairment (other than wearing glasses/contact lenses). They were paid at a rate of £8 per hour. Five participants were excluded based on the following criteria: (1) one for having missing trials in the vocabulary test, (2) one for having a mean response time shorter than 500 ms, (3) two for having more than 25% of the trials timed out, and (4) one for never selecting the first option during the entire experiment. No participants had a mean accuracy rate lower than the chance level (25%) or admitted to cheating during the task by looking up the meanings of the words. These data exclusion criteria were decided based on existing pilot data on a similar set of items before we analysed the primary data of Experiment 1, with the aim to exclude participants who were not fully engaged with the task (see Rodd, 2024 for discussion of this approach). Three participants had missing demographic or debriefing information due to a technical error, but their data of the vocabulary test were included for the stimulus optimisation due to low rates of exclusions in the rest of the sample. The final sample included 109 participants (82 females, 25 males, 1 preferring not to report, and 1 missing response), with at least 19 participants within each age band (i.e., 18–29, 30–39, 40–49, 50–59, 60–69). (See Table 1 for participants’ educational background).

**Table 1:** Experiment 1: Numbers of Participants with Different Education Levels for Each Age Band.


AGE BAND	HIGHEST EDUCATION LEVEL					

	NO FORMAL QUALIFICATIONS	SECONDARY SCHOOL/GCSE	COLLEGE/A LEVELS	UNDERGRADUATE DEGREE (E.G., BA/BSC)	GRADUATE DEGREE (E.G., MA/MSC)	DOCTORATE DEGREE (E.G., PhD)

18–29	0	1	11	7	0	0

30–39	0	10	3	14	0	1

40–49	0	2	7	8	4	1

50–59	1	4	7	3	4	0

60–69	0	6	4	7	3	0

Total	1	23	32	39	11	2


#### Materials

We selected 51 English words as target words with log-transformed frequency ranging from 0 to 8.85 in the SUBTLEX-UK database (van Heuven, Mandera, Keuleers, & Brysbaert, 2014). This ensured we included words at a wide range of difficulty levels. For each target word (e.g., *ubiquitous*), we selected a (near-)synonym (e.g., *omnipresent*) which shared a very similar meaning and word class. We then selected three semantically unrelated foils (e.g., *interpersonal, catatonic, voluminous*) based primarily on word class with approximately matched frequency and word length where possible. All words were selected to be relatively low in ambiguity, i.e., word forms were associated with a single meaning rather than multiple ones (Parks, Ray, & Bland, 1998; Rodd, Gaskell, & Marslen-Wilson, 2002). Note that participants’ performance on any given item is influenced not only by their vocabulary knowledge of the target word but also their vocabulary knowledge of the synonyms and foils. The synonyms and foils were mostly more frequent than the corresponding target words, but in some cases (especially with a high-frequency target, e.g., *collect*) the frequency of the synonym was lower than the target, such that participants’ performance may be limited by their knowledge of the synonym. (See Table 2 for descriptive statistics of stimuli properties and https://osf.io/p25jh for further details).

**Table 2:** Experiment 1: Descriptive Statistics (means and standard deviations) of Properties of Target Words, Synonyms, and Foils.


	LENGTH	FREQUENCY (RAW COUNTS)	FREQUENCY (PER MILLION)	FREQUENCY (LOG-TRANSFORMED)

Target	8.1 (1.87)	613.98 (1510.38)	3.05 (7.5)	3.99 (2.41)

Synonym	8.02 (2.35)	1523.04 (3310.76)	7.56 (16.43)	5.88 (1.87)

Foil	7.41 (1.97)	3297.65 (5475.68)	16.37 (27.18)	6.24 (1.98)


#### Procedure

The experiment was conducted online via Gorilla (Anwyl-Irvine et al., 2020, www.gorilla.sc). Participants confirmed their participation via a consent form and provided demographic details (e.g., gender, age, first/dominant language, bi-/multilingualism, highest education level, visual/cognitive/language-related impairment). Participants were required to use a laptop or desktop computer (not phone or tablet) to complete the experiment for the purpose of improving the precision and consistency of timing information (Rodd, 2024). (Response time was one of the dependent variables in the final vocabulary test, though it was not critical in Experiment 1.)

**Figure 1:**
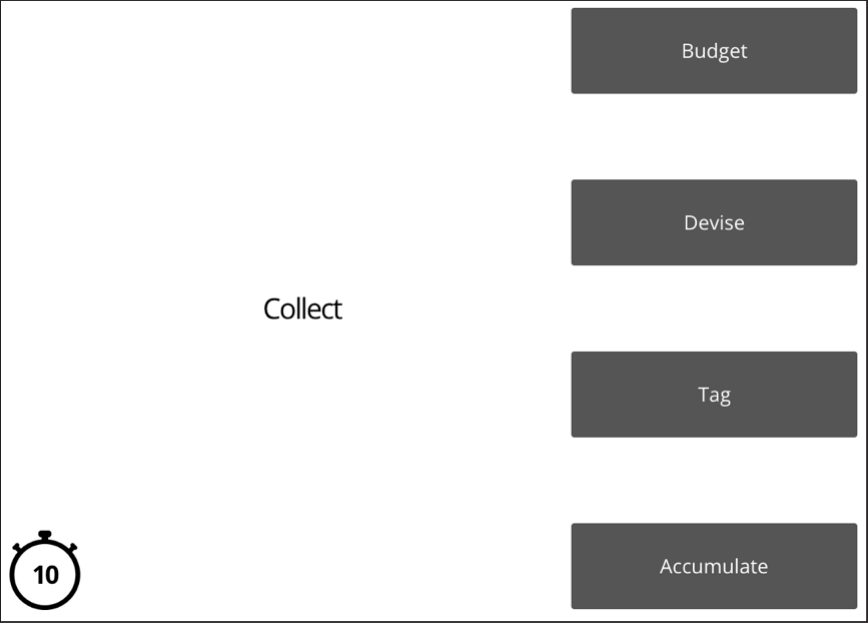
Experiment 1: Example trial.

In the vocabulary test, participants read the target word and four response options. They were asked to click on the word that was closest in meaning to the target from among the four options: one (near-)synonym and three unrelated foils. Participants had 10 seconds to respond. This time limit was included to reduce the possibility that participants could look up items online (Rodd, 2024). A countdown timer was displayed for 10 seconds or until response (see Figure 1 for an example trial). The four response options on each trial were presented in the same fixed order for all participants. Correct answers (i.e., synonyms) appeared in different pseudorandomised positions amongst the four response options on the screen across trials.

The experiment consisted of two experimental blocks. Trial order was randomised within each block and for each participant. While it is usual for tests designed to detect individual differences to use a single standardized order for all participants (Blott, Gowenlock, Kievit, Nation, & Rodd, 2023), in this case our primary aim was to select optimal items, so it was important that item performance was not systematically affected by their position in the list (e.g., boredom/practice effects). Participants could take a break between the two blocks for as long as they needed.

At the end of the experiment, participants were asked if they had cheated during the task by looking up the meanings of the words. They were told their answer would not affect payment and were encouraged to answer honestly.

### Results

Experiment 1 had one dependent variable: accuracy (1 = correct; 0 = incorrect). Fifty-three out of 5559 trials were timed-out with no responses and coded as incorrect. Prior to data analysis, we removed two words due to concerns that participants could select the correct answer based on knowledge of a higher frequency word with high orthographic/morphological overlap with the target word (*discomfit* similar to *discomfort*; *obfuscate* similar to *obscure*).

#### Item response theory analysis

**Figure 2:**
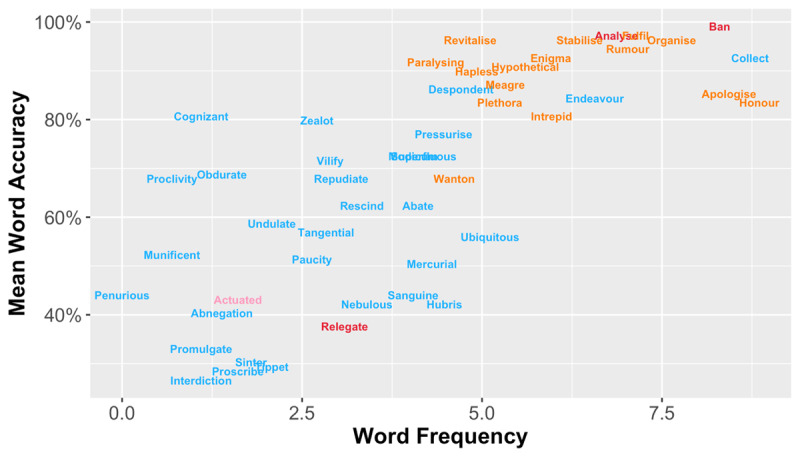
A scatter plot showing the relationship between log-transformed word frequency and mean accuracy for the final set of 30 words (blue) as well as items that were removed due to low correlation with participants’ overall performance (red), poor fit statistics of the Rasch model (orange), or unsatisfactory discriminability of the 2-PL logistic model (pink).

Following Yeatman et al. (2021), we conducted an IRT analysis based on participants’ accuracy data with the ‘MIRT’ package (version 1.35.1; Chalmers, 2012) in R software (version 4.0.2; R Core Team, 2020). First, we correlated participants’ correctness on individual word with their mean accuracy across all 49 words and removed three words that had a very low correlation coefficient (*r* < 0.1). Two of the removed words (i.e., *analyse, ban*) were quite simple and had very high accuracy, while the other (i.e., *relegate*) had a chosen synonym (i.e., *assign*) that was not close enough to its meaning resulting in relatively low accuracy (see red words in Figure 2).

We then aimed to remove words with unpredictable responses by fitting the Rasch model to the correctness responses of the 46 remaining words with a guess rate fixed at 0.25 based on the chance level for a 4-item multiple-choice question test. This step assessed how well real data and the model matched via two indices – infit and outfit – based on standardized residuals (i.e., the difference between real data points and model-predicted values divided by standard deviations). In general, values close to 1 indicate little data distortion. Fifteen words were removed as their infit or outfit values were outside a reasonable but lenient range between 0.6 and 1.4 (Aryadoust, Ng, & Sayama, 2020; Linacre, 2002; Wright & Linacre, 1994). Most of the removed words had relatively high frequency and accuracy (see orange words in Figure 2). Thirty-one words were retained and considered to have responses well-fitted by the Rasch model.

Finally, we examined item discrimination with the two-parameter logistic model (again with a guess rate fixed at 0.25) for the 31 remaining words and removed words that were poor at discriminating between participants in terms of their vocabulary knowledge. We followed the criterion used in Yeatman et al. (2021) and removed one word (i.e., *actuated*) that had a discriminability value slightly below the threshold 0.7 (see the pink word in Figure 2). This sequence of steps resulted in a final stimulus set of 30 items.

#### Reliability

We tested the internal consistency of the final set of 30 retained words by computing Cronbach’s α based on participants’ accuracy data on individual trials using the ‘CronbachAlpha’ function of the ‘DescTools’ package (version 0.99.44; Signorell, 2021) in R. Cronbach’s α is an index of internal consistency of a survey or test, i.e., how well all items in the survey or test measure a specific ability (Cronbach, 1951). As the accuracy data are dichotomous, Kuder-Richardson formula 20 (a.k.a. KR-20)^2^ was used for computing Cronbach’s α (Kuder & Richardson, 1937). The result revealed that the optimised set of 30 items reached a ‘good’ level of internal consistency (Cronbach’s α = 0.87, 95% CI = 0.86–0.87).

### Discussion

In Experiment 1, 109 participants who were aged between 18 and 69 performed the synonym-matching task including the initial set of 51 multiple-choice items. Based on participants’ accuracy, we excluded items that were poor at predicting vocabulary knowledge and had poor fit statistics of the Rasch model and unsatisfactory discriminability of the 2-PL logistic model. The final optimised subset of 30 items revealed a ‘good’ level of internal consistency, confirming the effectiveness of our IRT analysis.

## Experiment 2

Experiment 2 aimed to test the reliability and the validity of the newly developed 30-item vocabulary test in an independent sample of participants. There are several reasons why reliability might differ between Experiments 1 and 2. First, the reduction in test duration from 103 items to 30 items could potentially improve data quality due to reduced boredom/fatigue effects. Second, in Experiment 2 we presented items in a single fixed order to all participants to ensure that all participants experienced the task in an identical manner such that any variability in order effects across participants was minimised (Blott et al., 2023; Swets, Desmet, Hambrick, & Ferreira, 2007). In addition, we made some minor changes to the procedure to further improve the smooth running of the task and added five very easy target-synonym pairs (e.g., *big* – *large*) that could be used by future researchers as attention checks to exclude participants (Rodd, 2024). Finally, it is possible that reliability may be reduced in Experiment 2 on this subset of items as for Experiment 1 the IRT procedure will have selected items that were optimised for the particular idiosyncratic data provided by that particular set of participants.

To examine the validity of the test, we explored the impact of two factors that are well known to affect vocabulary knowledge. First, we predicted a significant effect of word frequency, with higher word frequency associated with higher accuracy rates and shorter response times. Moreover, in light of the evidence for an accumulation of vocabulary knowledge with advancing age (Hoffman, 2018; Miller et al., 2009; Park et al., 2002; Verhaeghen, 2003), we also predicted that age would modulate accuracy rates. Specifically, we expected a significant effect of age, with increasing age associated with higher accuracy rates. We did not expect any specific age effects on response times as ageing may cause opposite effects on the time for synonym matching. On the one hand, older adults could be faster than younger adults because they have presumably more vocabulary knowledge. On the other hand, older adults could be slower than younger adults given that their performances on a number of cognitive tasks were found slower than their younger counterparts (Salthouse, 1996). The absence of ageing effect on response times was also observed in a previous study using the synonym judgement task (Hoffman, 2018).

Prior to data collection, we preregistered this experiment (including our materials, procedure, data exclusion criteria, hypotheses, and predictions) through the OSF (https://osf.io/we9bg). Materials, data, and analysis scripts for Experiment 2 are available here: https://osf.io/mgsdq/.

### Method

#### Participants

We aimed to recruit 120 participants through Prolific (www.prolific.co) with an equal number of participants in six age bands (i.e., 18–29, 30–39, 40–49, 50–59, 60–69, and 70–79). Our sample size was determined based on the sample size of a recent study wherein 120 participants were assessed on reading ability online (Yeatman et al., 2021). We pre-screened participants based on the following criteria: (1) aged 18–79 years, (2) located in the UK, (3) British English as first language, (4) monolingual, (5) no diagnosis of dyslexia or another reading or language disorder, (6) no diagnosis of mild cognitive impairment or dementia, (7) no visual impairment (other than wearing glasses/contact lenses), and (8) approval rate no less than 80% on Prolific. Participants were recruited separately for each age band on Prolific and were paid for participating at a rate of £8 per hour. Two participants were excluded and replaced because they reported inconsistent information about age and first language in our demographic questionnaire and their profile on Prolific. We accidentally over-recruited by one participant, yielding 121 participants (83 females and 38 males) in the final sample with 21 participants for the 18–29 age band and 20 participants for the others. (See Table 3 for participants’ educational background).

**Table 3:** Experiment 2: Numbers of Participants with Different Education Levels for Each Age Band.


AGE BAND	HIGHEST EDUCATION LEVEL					

	NO FORMAL QUALIFICATIONS	SECONDARY SCHOOL/GCSE	COLLEGE/A LEVELS	UNDERGRADUATE DEGREE (E.G., BA/BSC)	GRADUATE DEGREE (E.G., MA/MSC)	DOCTORATE DEGREE (E.G., PhD)

18–29	0	0	12	8	1	0

30–39	0	3	5	11	1	0

40–49	0	0	9	8	2	1

50–59	0	8	6	5	0	1

60–69	0	4	6	7	2	1

70–79	1	4	10	4	1	0

Total	1	19	48	43	7	3


#### Materials

The thirty low-ambiguity English words selected in Experiment 1 were used as target words in the present experiment. The words’ log-transformed frequency ranged from 0 to 8.72 in the SUBTLEX-UK database (van Heuven et al., 2014) and their estimated difficulty ranged from –1.74 to 3 based on the 2-PL IRT analysis reported in Experiment 1. The (near-)synonym and three foils for each target word remained the same as in the Experiment 1, except that the original (near-)synonym for the target word *interdiction* was changed due to its ambiguity (original synonym: *sanction*; new synonym: *ban*). An additional five simple words with relatively early age of acquisition (e.g., target: *big* – synonym: *large*) were added and evenly distributed throughout the experiment as attention checks. (See Table 4 for descriptive statistics of stimuli properties and https://osf.io/tgx6a for further details).

**Table 4:** Experiment 2: Descriptive Statistics (means and standard deviations) of Properties of Target Words, Synonyms, and Foils.


	LENGTH	FREQUENCY (RAW COUNTS)	FREQUENCY (PER MILLION)	FREQUENCY (LOG-TRANSFORMED)

Target	8.4 (1.77)	260.33 (1118.23)	1.3 (5.55)	3.02 (1.9)

Synonym	7.77 (2.71)	1184.77 (2476.69)	5.88 (12.29)	5.62 (1.95)

Foil	7.41 (2.03)	2304.08 (3704.84)	11.44 (18.39)	5.97 (1.97)


#### Procedure

**Figure 3:**
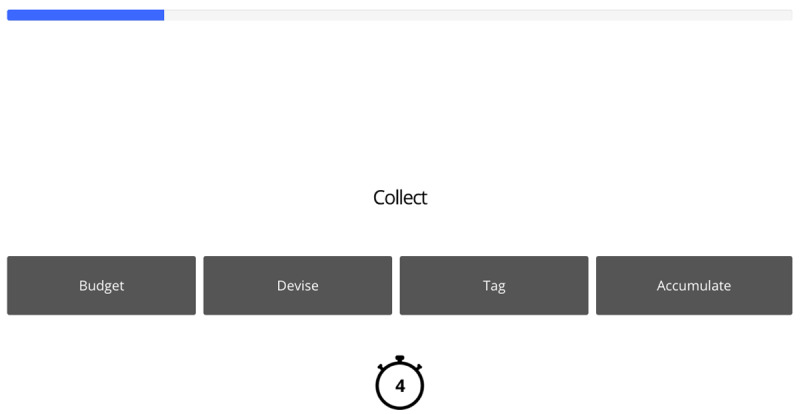
Experiment 2: Example trial.

The procedure of Experiment 2 was identical to Experiment 1, except for the following modifications: (1) the four response options were presented horizontally rather than vertically for a more intuitive layout consistent with the left-to-right reading direction in English (see Figure 3 for an example trial), (2) no more than two consecutive trials had their correct answers (i.e., synonyms) appearing in the same position on the screen to reduce accidental mis-clicking, (3) the countdown timer was invisible until four seconds were left to reduce distraction, (4) a progress bar was added to help participants get a sense of how long the task would be and to help them avoid ending the task near completion, and (5) trial order was kept the same for all participants to minimise differences in task experience among participants (Swets et al., 2007, p. 67).

#### Data analysis

The experiment had one within-participants continuous independent variable (word frequency) and one between-participants continuous independent variable (age). The experiment had two dependent variables: accuracy (1 = correct; 0 = incorrect) and response time (in milliseconds). Thirty-six out of 4235 trials were timed-out with no responses and coded as incorrect. Our primary index for the measurement of vocabulary knowledge was participants’ accuracy rates, i.e., the proportion of correct trials. Test reliability, i.e., internal consistency, was assessed via Cronbach’s α as in Experiment 1.

Each target word’s raw frequency count in the SUBTLEX-UK database was log-transformed. Words with a zero frequency were assigned a raw frequency score of 1 for the purpose of log-transformation. Both word frequency and age were centred using the ‘scale’ function (version 4.0.2; Becker, Chambers, & Wilks, 1988) in R. Response time data were log10-transformed to meet the required assumptions of normality and homoscedasticity for the analysis (confirmed through inspection of histograms and scatterplots of fitted values vs. residuals).

The accuracy and response time (correct trials only) data were analysed with logistic and linear mixed-effects models with crossed random effects for participants and items (Baayen, Davidson, & Bates, 2008). To fit the models, we used the ‘glmer’ function for the binary accuracy data and the ‘lmer’ function for the response time data from the ‘lme4’ package (version 1.1-27.1; Bates, Mächler, Bolker, & Walker, 2015) in R. We followed recommendations by Barr, Levy, Scheepers, and Tily (2013) to fit the models with maximal random effect structures. The maximal model included fixed effects for word frequency and age, the frequency-by-age interaction, by-participants random effects with an intercept and a slope for word frequency, and by-item random effects with an intercept and a slope for age. Where the maximal model did not converge, we took the following steps in turn until a model and its reduced models with the factors of interest removed converged: (1) the correlations between the random intercepts and the random slopes were removed, (2) the random intercepts were removed while retaining the random slopes, (3) a data-driven forward model selection approach was adopted, starting with the simplest model with only random intercepts and adding the random slopes one by one. Likelihood ratio tests (α = .2) were used to test whether any models with the random slopes added showed a significant improvement on the simplest model (Barr et al., 2013; Matuschek, Kliegl, Vasishth, Baayen, & Bates, 2017). If none of these models showed a significant improvement, then the simplest model with only random intercepts was used as the final model for the analysis. If multiple models showed a significant improvement, then the model with the smallest p-value was taken first and compared to models containing this slope and another slope that had a significant improvement one at a time to see if there was any further improvement. This procedure continued until there was no significant improvement and the final model was determined. If the random effects highly correlated with one another (i.e., the estimate of correlation was exactly –1 or 1, which was a sign of model overfitting), the next best model that converged (with the most complex random effects structure) was used instead. Our final model for both accuracy and response time was as follows: Accuracy/Response time ~ 1 + centred log-transformed word frequency + centred age + centred log-transformed word frequency:centred age + (1 | participant) + (1 + centred age | item).

Likelihood ratio tests (α = .05) were used to test whether the fixed effects were significant by comparing the final model to reduced models with each factor of interest removed in turn (with the rest of the model structure intact).

No participants’ data were excluded from the above analysis based on the following preregistered criteria: (1) failing to complete the experiment or having an incomplete dataset, (2) admitting to cheating during the task by looking up the meanings of the words, or (3) failing to pay attention to the task (more than one attention check trial answered incorrectly). Two participants happened to answer incorrectly on only one and the same attention check trial but selected different foils likely due to an attention lapse or mis-clicking. In addition, trials with a response time shorter than 500 ms were excluded from both the accuracy analysis and the response time analysis. (Here the accuracy analysis deviated from the preregistration to be consistent with the response time analysis; however, only 3 out of 3630 observations were removed from the accuracy analysis due to extremely short response times.) These data exclusion criteria were slightly modified from Experiment 1 to consider the five simple attention check trials and the response time analysis that had been added in Experiment 2. We decided and preregistered these criteria before data collection.

### Results

#### Reliability

Cronbach’s α based on participants’ accuracy rates was 0.82 (95% CI = 0.81–0.83). This is somewhat reduced compared to Experiment 1 (α = 0.87), perhaps reflecting the fact that our IRT procedure to select items was based on performance of a different sample in Experiment 1. Importantly, 0.82 still reflects a ‘good’ level of internal consistency (α ≥ 0.8; George & Mallery, 2019, p. 244).

#### Accuracy

**Figure 4:**
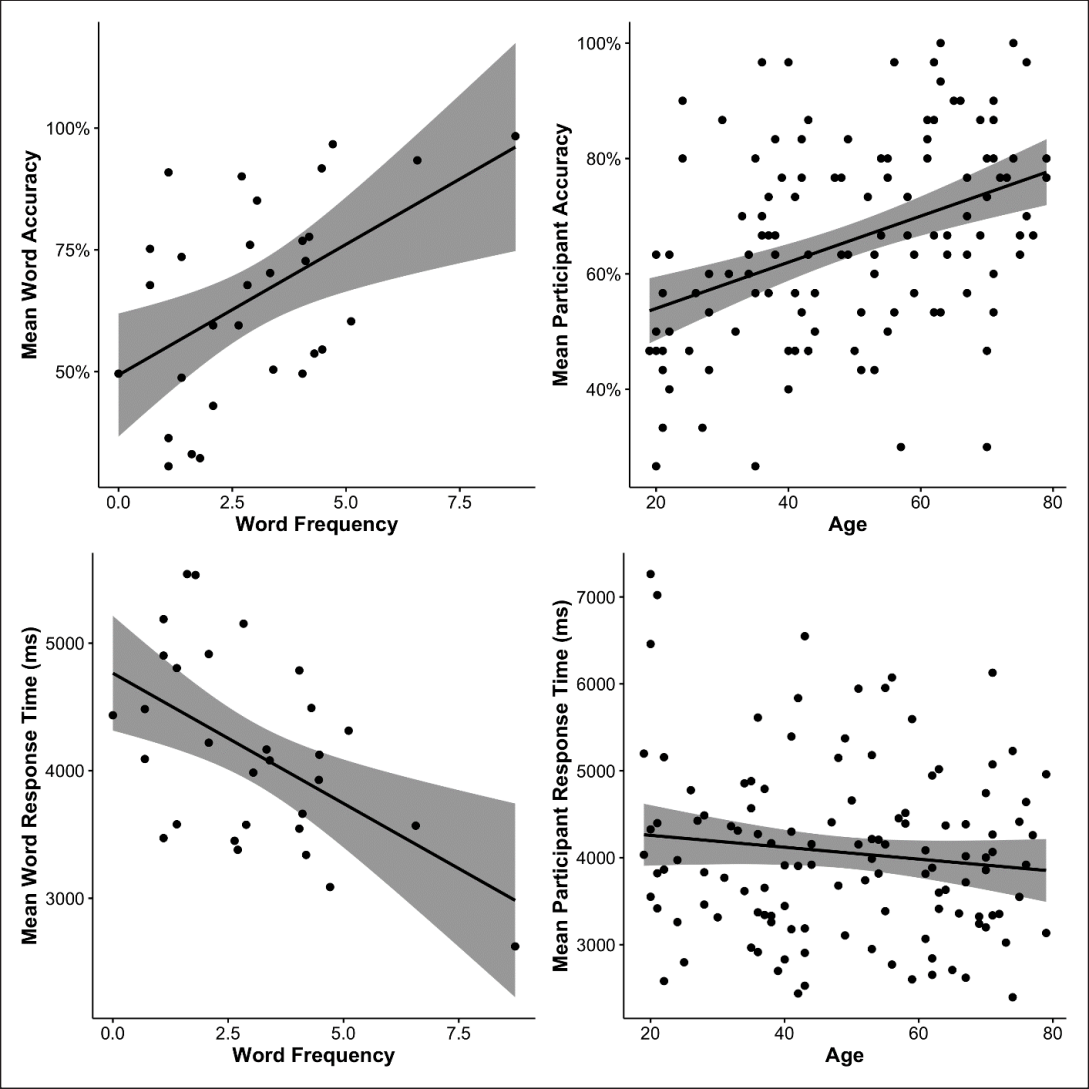
Scatter plots showing the relationships between task performances (accuracy rate; response time) and predictor variables (log-transformed word frequency; age by year). For both left-hand panels, each dot represents a target word. For both right-hand panels, each dot represents a participant. All panels were plotted with a linear regression line and 95% confidence interval (i.e., the shaded area).

Table 5 shows descriptive statistics of participants’ accuracy rates for each age band. Participants’ accuracy rates spanned a wide range in each age band, indicating that the items in our test were not too easy or too hard for any age band. No participant showed an accuracy rate below the chance level (25%). The lowest accuracy rate in the current sample was 26.67% (i.e., 8 correct trials out of 30 critical trials) and observed in only 2 relatively younger participants. The highest accuracy rate in the current sample was 100% and observed in only 2 relatively older participants. Accuracy rates became higher when word frequency increased (see Figure 4, top-left panel) and when age increased (see Figure 4, top-right panel). These patterns were confirmed by a significant effect of word frequency, *χ*^2^(1) = 11.17, *p* < .001, and a significant effect of age, *χ*^2^(1) = 15.93, *p* < .001. There was no significant frequency-by-age interaction, *χ*^2^(1) = 0.06, *p* = .800.

**Table 5:** Descriptive Statistics (means, standard deviations, minimums, and maximums) of Accuracy and Response Time for Each Age Band.


AGE BAND	ACCURACY (%)				RESPONSE TIME (MS)			
	
	MEAN	SD	MIN	MAX	MEAN	SD	MIN	MAX

18–29	51.19	14.79	26.67	90.00	4385.80	1258.44	2580.94	7262.89

30–39	67.17	14.72	26.67	96.67	3902.85	786.70	2697.78	5612.14

40–49	64.48	16.16	42.86	96.67	4009.75	1147.89	2438.48	6548.14

50–59	61.83	15.65	30.00	96.67	4337.30	1020.83	2600.48	6073.26

60–69	76.50	14.73	53.33	100.00	3634.65	702.30	2619.40	5018.37

70–79	72.67	16.67	30.00	100.00	4077.52	896.66	2394.80	6127.77


#### Response time

Table 5 shows descriptive statistics of participants’ response times for each age band. Response times became shorter when word frequency increased (see Figure 4, bottom-left panel). Age did not modulate participants’ response times (see Figure 4, bottom-right panel). These patterns were confirmed by a significant effect of word frequency, *χ*^2^(1) = 10.99, *p* < .001. There was no significant effect of age, *χ*^2^(1) = 0.25, *p* = .620. The frequency-by-age interaction did not reach statistical significance in the current dataset, *χ*^2^(1) = 2.92, *p* = .088.

### Discussion

In Experiment 2, the optimised subset of 30 multiple-choice items selected via the IRT analysis reported in Experiment 1 was tested in a new sample of 121 participants. The subset of 30 items reached a ‘good’ level of internal consistency. The data replicated well-established positive effects of word frequency and age on participants’ accuracy. Participants’ response times reduced significantly with increasing word frequency but were not modulated by age likely due to opposite effects of ageing on their response times – compared to young participants, older participants could be faster because of the help of richer vocabulary knowledge but could be slower due to the generalized slowing of cognitive processing (Salthouse, 1996).

## General discussion

The field of psycholinguistics has a growing need for vocabulary tests that can be administered quickly and easily online owing to the proliferation of web-based experiments. This study presents the WORDS test – a new easy to implement, reliable, quick, web-based, and open-access assessment of English vocabulary knowledge. In this test, participants’ vocabulary knowledge is assessed via a synonym-matching task. The optimal test items were selected by conducting an IRT analysis based on participants’ accuracy data in Experiment 1. The reliability of this newly developed vocabulary test was good (Cronbach’s α = 0.82), which was assessed based on accuracy data from a new sample in Experiment 2. We also verified the test’s validity by confirming its ability to show the well-replicated findings that (1) more frequent words were responded to more accurately and quickly compared to less frequent words, and (2) greater vocabulary knowledge (indexed by higher accuracy rates) was associated with older compared to younger age. The test has 30 test items and takes on average 4 minutes (5 minutes 40 seconds including consent/debrief) to complete.

These findings jointly suggest that this newly developed web-based vocabulary test is not only useful but also short enough to be easily implemented in any online experiment to reliably assess English vocabulary knowledge in both younger and older adults. Accuracy on 5 additional attention check items can be used by future researchers to exclude participants based on inadequate attention levels. The WORDS test could be helpful for researchers interested in the relationship between individuals’ vocabulary knowledge and other cognitive abilities (e.g., IQ, executive functions, learning of new words, or statistical learning, etc) or used as a screen to ensure that all participants reach some minimum level of English proficiency. As native speakers range from low to high levels of vocabulary knowledge, we suggest that researchers determine for themselves what level of vocabulary knowledge is required for their particular research goals. All materials, data, and analysis scripts are available via the OSF (https://osf.io/mgsdq/). The task can be freely accessed via Gorilla Open Materials (https://app.gorilla.sc/openmaterials/694887), which ensures that the procedure of future uses of the task will be identical, thus allowing for useful comparisons between data collected by different researchers. As the WORDS test is primarily developed as a research tool (not as a web app for the general public), task performance is not directly reported to participants at the end of the task. However, raw data are recorded and saved on Gorilla (www.gorilla.sc) for experimenters to access, and correct answers are also provided on the OSF (https://osf.io/tgx6a). Lastly, this work can also serve as an effective protocol for researchers who are interested in developing a quick, reliable, and web-based vocabulary test in other languages.

We note a few limitations of this study. First, although we confirmed that the WORDS test showed the well-replicated frequency and age effects on participants’ task performances, its relationships with other English vocabulary tests (e.g., The Peabody Picture Vocabulary Test, The Vocabulary Size/Levels Test, The Mill Hill vocabulary scale, etc.) were not examined in this study. In addition, the WORDS test was originally developed for assessing English native speakers’ vocabulary knowledge. In the current sample of native speakers, we observed that 2 relatively younger participants’ accuracy was very near chance despite passing the attention check, and that 2 relatively older participants’ accuracy was at ceiling. Given such a small number of participants showing the extreme performance, more data is needed for better understanding of the reliability of the test at these extremes. The test’s suitability to assess bilingual speakers’ and ESL learners’ English vocabulary knowledge or proficiency is still unclear. Caution is advised when using the WORDS test for bilingual speakers and ESL learners across different proficiency levels. For instance, the LexTALE (Lemhöfer & Broersma, 2012) is more suitable for advanced learners of English than those with lower proficiency (Liu & Chaouch-Orozco, 2024; Puig-Mayenco, Chaouch-Orozco, Liu, & Martín-Villena, 2023). Future work may explore these issues by assessing the concurrent validity of the WORDS test and other English vocabulary or proficiency tests in both native and non-native samples at all levels of English vocabulary knowledge (e.g., Harel et al., 2024).

## Data Accessibility Statement

All materials, data, and analysis scripts are available on the OSF (https://osf.io/mgsdq/). Experiment 2 was preregistered before data collection via the OSF (https://osf.io/we9bg) and the experimental task can be freely accessed via Gorilla Open Materials (https://app.gorilla.sc/openmaterials/694887).

## Footnotes

The test’s reliability as assessed by Cronbach’s α is considered to be: excellent if larger than or equal to 0.9, good if larger than or equal to 0.8 but smaller than 0.9, acceptable if larger than or equal to 0.7 but smaller than 0.8, questionable if larger than or equal to 0.6 but smaller than 0.7, poor if larger than or equal to 0.5 but smaller than 0.6, or unacceptable if smaller than 0.5 (George & Mallery, 2019, p. 244).

KR-20 = (k / (k–1)) * (1 – Σp_j_q_j_ /σ^2^) where: k = Total number of items; p_j_ = Proportion of individuals who answered item j correctly; q_j_ = Proportion of individuals who answered item j incorrectly; σ^2^ = Variance of scores (i.e., numbers of correct responses) for all participants.
